# Computational Fluid Dynamics Modelling of Hydrogen Production via Water Splitting in Oxygen Membrane Reactors

**DOI:** 10.3390/membranes14100219

**Published:** 2024-10-17

**Authors:** Kai Bittner, Nikolaos Margaritis, Falk Schulze-Küppers, Jörg Wolters, Ghaleb Natour

**Affiliations:** 1 Central Institute of Engineering, Electronics and Analytics (ZEA), Forschungszentrum Jülich GmbH, 52425 Jülich, Germany; 2 Faculty of Mechanical Engineering, RWTH Aachen University, 52056 Aachen, Germany; 3 ISF, Faculty of Mechanical Engineering, RWTH Aachen University, 52056 Aachen, Germany

**Keywords:** hydrogen production, membrane reactor, oxygen transport membrane, water splitting, computational fluid dynamics

## Abstract

The utilization of oxygen transport membranes enables the production of high-purity hydrogen by the thermal decomposition of water below 1000 °C. This process is based on a chemical potential gradient across the membrane, which is usually achieved by introducing a reducing gas. Computational fluid dynamics (CFD) can be used to model reactors based on this concept. In this study, a modelling approach for water splitting is presented in which oxygen transport through the membrane acts as the rate-determining process for the overall reaction. This transport step is implemented in the CFD simulation. Both gas compartments are modelled in the simulations. Hydrogen and methane are used as reducing gases. The model is validated using experimental data from the literature and compared with a simplified perfect mixing modelling approach. Although the main focus of this work is to propose an approach to implement the water splitting in CFD simulations, a simulation study was conducted to exemplify how CFD modelling can be utilized in design optimization. Simplified 2-dimensional and rotational symmetric reactor geometries were compared. This study shows that a parallel overflow of the membrane in an elongated reactor is advantageous, as this reduces the back diffusion of the reaction products, which increases the mean driving force for oxygen transport through the membrane.

## 1. Introduction

**Figure 1:**
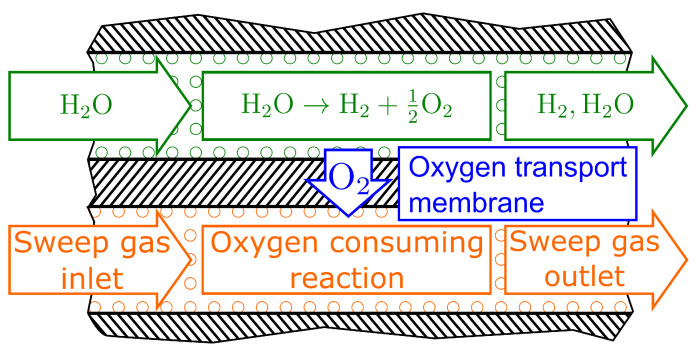
Schematic illustration of water splitting in oxygen membrane reactors.

The integration of oxygen transport membranes into reactors enables the coupling of chemical reactions with separation processes, allowing for the development of new efficient reactor concepts. In this context, a feed gas is separated from a sweep gas by a membrane, conducting oxygen ions as well as electrons. By establishing a difference in chemical potential, oxygen is thus transported from the feed to the sweep side. The water splitting process, illustrated schematically in Figure 1, in which $H_{2}O$ serves as the feed gas, is of particular interest. By removing oxygen, pure hydrogen can be produced. To maintain the chemical potential difference, typically, a reducing gas is used on the sweep side. The feasibility of this concept has been experimentally demonstrated in several previous studies [1,2,3,4,5,6,7,8]. Methane, carbon monoxide, and hydrogen were considered as reducing gases in these studies. By using methane, the water splitting reaction can be coupled with the partial oxidation of methane, producing both high-purity hydrogen and synthesis gas with an approximate $H_{2}$:CO ratio of 2:1 suitable for certain downstream processes such as methanol synthesis [9]. This makes this reactor concept a potential alternative to steam methane reforming. Pure hydrogen is mostly used for experimental studies to establish the required gradient in chemical potential since it does not yield net hydrogen production. A membrane reactor may, however, be used as an energy-efficient hydrogen purification process (e.g., for coke oven gas) [7]. Similarly, mixtures with high CO content such as product streams from coal gasification may be utilized for the production of high purity $H_{2}$ [2].

For a deeper understanding and an effective design optimization of these reactors, mathematical models are needed. In a prior study, we presented a perfectly mixed reactor model assuming chemical equilibrium for initial design iterations and validated it against experimental data [10]. However, the model from the mentioned study does not consider the reactor geometry and does not allow us to investigate the influence of diffusion processes in the gas phase or temperature distribution inside the reactor. This limitation can be addressed through computational fluid dynamics (CFD), which allows for the modelling of complex geometries and is widely used in the field of reactor modelling [11,12,13,14,15,16]. In CFD simulations of water splitting using oxygen transport membranes in previous studies [17,18], instead of $H_{2}O$, a mixture of $H_{2}$ and $O_{2}$ with a ratio of 2:1 was used.

This study presents an approach to the CFD modelling of water splitting inside oxygen membrane reactors by assuming oxygen transport through the membrane to be the rate-determining process. For this purpose, the local oxygen flux through the membrane is calculated by the Wagner equation using the chemical equilibrium oxygen partial pressure on the membrane surfaces. On the feed side, the local generation of hydrogen and the consumption of steam are modelled by source terms calculated from the stoichiometry of the reaction and the oxygen flux. On the sweep side, an oxygen-consuming reaction is modelled in a similar way. The reaction heat is modelled by an energy source term calculated from the enthalpy of formation.

The simulations do not require knowledge of surface reaction rates, as the hydrogen production is directly determined from the oxygen flux. The model is validated against experimental data from the literature [5] for a rotational symmetric geometry using hydrogen as the reducing gas. The simulated hydrogen production rate and the simulated oxygen partial pressure at the outlet of the reactor are compared with measured data. Furthermore, a simulation study demonstrating how the model can be utilized to optimize reactor design is conducted. While the CFD model can be applied to complex geometries, only rotational symmetric and 2-dimensional geometries are modelled and compared in this study for the sake of simplicity. In addition to hydrogen, methane is also considered as a reducing gas, whereby a chemical equilibrium at the membrane surface is assumed as a first approximation, assuming fast reaction kinetics.

## 2. Materials and Methods

### 2.1. CFD Modelling

The CFD modelling was performed using the commercial finite volume solver Ansys Fluent 2023R2. For this study, the code was extended to model the membrane.

#### 2.1.1. Fluid Modelling

The main equations that were solved in the CFD simulation are briefly discussed below. For this study, the so-called source terms in the equations are particularly important, as these terms are used to model the membrane. The source terms are defined in Section 2.1.2.

The fluid flow is modelled using the Navier–Stokes equation for stationary laminar flow [19]:

(1) $$\nabla \cdot \left(\rho v\right)=S_{m}$$

(2) $$\nabla \cdot \left(\rho vv\right)=-\nabla P+\nabla \cdot \left(\mu \left[\left(\nabla v+\nabla v^{T}\right)-\frac{2}{3}\nabla \cdot vI\right]\right)$$

Here, ρ is the density of the gas mixture calculated by the ideal gas law, whereby the change in density was only modelled as a function of temperature due to the small pressure differences in the reactor. v is the velocity vector, *P* is the pressure, μ is the shear viscosity, and *I* is the unit tensor. The term $S_{m}$ on the right-hand side of the continuity equation is the mass source used to describe the mass transport through the membrane, which is defined in Equation (11).

The local mass fractions of each species are modelled using the species conservation equation, which takes the form for species *i* [19]:

(3) $$\nabla \cdot \left(\rho vY_{i}\right)=-\nabla \cdot J_{i}+S_{i}$$

$Y_{i}$ is the mass fraction, $J_{i}$ is the diffusion flux, and $S_{i}$ is the species source used to describe the creation and elimination of species due to reactions on the membrane surface and is defined in Equation (12). It should be noted here that in this study, oxygen is not modelled as a separate species in the CFD simulation, but its partial pressure is determined from chemical equilibrium (c.p. Section 2.1.2). This is based on the assumption that the oxygen content in the water vapour is negligible with a partial pressure in the order of $10^{-6}$ atm at an operating temperature of (850 °C to 950 °C in this study) calculated via Cantera 3.0 [20].

This diffusion flux was modelled by Ficks law using mixture diffusion coefficients calculated by the mixture-averaged evaluation as [21]

(4) $$D_{i,m}={\left(\sum_{j\neq i}\frac{X_{j}}{D_{ij}}+\frac{X_{i}}{1-Y_{i}}\sum_{j\neq i}\frac{Y_{j}}{D_{ij}}\right)}^{-1},$$

where $X_{j}$ is the mole fraction and $D_{ij}$ is the binary diffusion coefficient. This methodology is an approximation to the more rigorous but also more computationally expensive Stefan–Maxwell formulation, which resulted in an error of about 0.1% for the simulated hydrogen production rate during testing. The binary diffusion coefficients were obtained from the Chapman–Enskog formula [22].

The temperature distribution inside the reactor is predicted using the energy conservation equation. Neglecting viscous dissipation, it reads [19]

(5) $$\nabla \cdot \left[v\left(\rho \left[h_{sens}+\frac{{\left|v\right|}^{2}}{2}\right]+P\right)\right]=\nabla \cdot \left(k\nabla T-\sum_{i}h_{sens,i}J_{i}\right)$$

Here, $h_{sens}$ is the sensible enthalpy of the mixture, *k* is the thermal conductivity of the mixture, *T* is the temperature, and the term $h_{sens,i}J_{i}$ is the diffusion energy flux for species *i*. For solid regions such as the membrane, the equation simplifies to

(6) $$\nabla \cdot \left(k\nabla T\right)+S_{h}+S_{rad}=0$$

Here, $S_{h}$ is the energy source due to the reactions taking place on the membrane surface, which is defined in Equation (13), and $S_{rad}$ is the energy source due to radiation calculated from the Stefan–Boltzmann law by assuming the membrane as a grey body.

As boundary conditions for the inlets on the feed and sweep side, mass flow rates are defined. These are calculated from the molar flow rates as

(7) $${\dot{m}}_{in}={\dot{N}}_{in}{\bar{M}}_{in},$$

where ${\bar{M}}_{in}$ is the mean molar mass. The corresponding mass fractions $Y_{i}$ are calculated from the specified gas mole fractions at the inlets $X_{in,i}$ as

(8) $$Y_{in,i}=X_{in,i}\frac{M_{i}}{{\bar{M}}_{in}}.$$

The temperature at the inlet is set equal to the operating temperature of the reactor. At the outlets, pressure outlets with a gauge pressure of $P_{gauge}=0$ Pa are defined. Reverse flow into the domain is not permitted in the present study. At the fluid–wall interface, no-slip conditions are applied, and the fluid temperature is set equal to the wall temperature, i.e., v=0 and $T_{fluid}=T_{wall}$. At the outer wall boundaries of the computational domain, the temperature is set equal to the operating temperature of the reactor.

#### 2.1.2. Membrane Modelling

The membrane is modelled by including source terms for mass, species, and energy into Equations (1), (3), and (6), respectively. This is achieved by extending the CFD code with a User-Defined Function (UDF).

**Figure 2:**
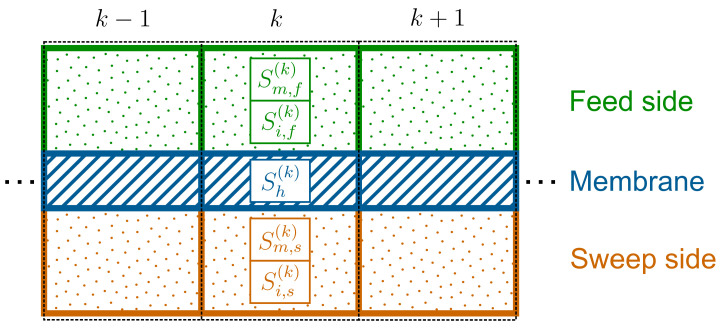
Representation of the mesh of the membrane and the cells on its surface including the source terms.

In *Ansys Fluent*, the governing field equations are iteratively solved using the finite volume method. Herein, the domain is discretized into a finite number of computational cells called a mesh. Cell-centered values for the solution variables are available for each cell in this mesh. At each iteration, the UDF accesses these values and returns the corresponding source terms. For this purpose, the mesh of the membrane is modelled such that it consists of only one cell in thickness direction with an equal area facing the feed and the sweep side as shown in Figure 2. Note that while in the figure only the computational fluid cells adjacent to the membrane are shown, the entire reactor geometry can be discretized into computational cells, allowing for the investigation of flow, diffusion, and heat transfer processes within complex reactor geometries. In contrast to modelling the membrane as a 2D surface in the simulation, the representation of it by solid cells allows for the space occupied by the membrane to be taken into account, which may be important for complex reactor geometries. Based on the values of the cell-centred variables, the required source terms for the cells are then calculated.

Assuming oxygen transport through the membrane to be the rate-determining process for the water splitting reaction, the oxygen flux through an oxygen transport membrane can be calculated using the Wagner equation [23,24]. Furthermore, given this assumption, the driving force can be estimated from the chemical equilibrium oxygen partial pressure [10], which is independent of reaction rates and, thus, also of the catalyst. The local oxygen flux through the membrane cell *k* in mol·m^−2^·s^−1^ is, therefore, given by

(9) $$j_{O_{2}}^{\left(k\right)}=\frac{R}{16F^{2}}\frac{{\bar{\sigma }}_{amb}T^{\left(k\right)}}{L}\ln\frac{p_{O_{2},eq,f}^{\left(k\right)}}{p_{O_{2},eq,s}^{\left(k\right)}}.$$

Here, *R* is the universal gas constant, ${\bar{\sigma }}_{amb}$ is the average ambipolar conductivity, *F* is the Faraday constant, *L* is the thickness of the membrane, and $p_{O_{2},eq}$ is the chemical equilibrium oxygen partial pressure. The subscripts *f* and *s* denote the feed and sweep side, respectively. For the calculation of $p_{O_{2},eq}$, an internal *Ansys Fluent* function is used, which returns the mass fractions at a chemical equilibrium for a set of species. The chemical equilibrium oxygen partial pressure is calculated from this as

(10) $$p_{O_{2},eq}^{\left(k\right)}=Y_{O_{2},eq}^{\left(k\right)}\frac{{\bar{M}}^{\left(k\right)}}{M_{O_{2}}}P^{\left(k\right)}\;,$$

where $Y_{O_{2},eq}$ is the equilibrium oxygen mass fraction, $M_{O_{2}}$ is the molar mass of oxygen, and ${\bar{M}}^{\left(k\right)}$ is the mean molar mass of the mixture.

From the local oxygen flux, the local mass source in Equation (1) is computed as

(11) $$S_{m,f/s}^{\left(k\right)}=\pm \frac{A^{\left(k\right)}}{V_{f/s}^{\left(k\right)}}j_{O_{2}}^{\left(k\right)}M_{O_{2}}.$$

$A^{\left(k\right)}$ is the area of the *k*-th membrane cell facing the feed/sweep side, and $V_{f/s}^{\left(k\right)}$ is the volume of the computational cell to which the mass source is added. The term is negative on the feed side, as mass is removed, and positive on the sweep side, as mass is added.

The species source terms for Equation (3) are calculated in a similar way. Assuming all of the released oxygen due to water splitting on the feed side is consumed on the sweep side, the source terms can be written as

(12) $$S_{i,f/s}^{\left(k\right)}=\frac{A^{\left(k\right)}}{V_{f/s}^{\left(k\right)}}j_{O_{2}}^{\left(k\right)}\frac{v_{i,f/s}}{|v_{O_{2},f/s}|}M_{i}.$$

Here, the stoichiometric numbers $v_{i}$, which are defined below, are included to take the stoichiometry of the surface reactions into account. Note that since oxygen is not modelled as a separate species, there is no source term for it.

For the energy source, it is assumed here that the chemical reaction takes place directly on the surface of the membrane. Furthermore, it is assumed that the temperature difference between the membrane surface on the feed side and the sweep side is negligible due to the low membrane thickness. Therefore, the energy source is calculated from the net enthalpy change due to reactions on the feed and sweep side. It is included in the energy equation for the membrane domain (Equation (6)). The energy source term for membrane cell *k* then reads

(13) $$S_{h}^{\left(k\right)}=-\frac{A^{\left(k\right)}}{V_{m}^{\left(k\right)}}j_{O_{2}}^{\left(k\right)}\sum_{i}\left(\frac{v_{i,f}}{|v_{O_{2},f}|}+\frac{v_{i,s}}{|v_{O_{2},s}|}\right)h_{i}^{0},$$

where $h_{i}^{0}$ is the enthalpy of formation of species *i*. Note that while the energy source term cancels out when hydrogen is used as a sweep gas, it is included here to ensure the model’s applicability to other reactions. As it is demonstrated in Section 3.2.3, using methane as a sweep gas leads to a cooling of the membrane due to a net endothermic reaction. The corresponding stoichiometric numbers for the surface reactions can be calculated from the stoichiometry of the reactions. For water splitting, the reaction can be written as

(14) $$H_{2}O\to H_{2}+\frac{1}{2}O_{2}.$$

The non-zero stoichiometric numbers are $v_{H_{2}O,f}=-1$, $v_{H_{2},f}=1$, and $v_{O_{2},f}=\frac{1}{2}$.

One way to maintain the difference in chemical potential between the feed and the sweep side is to use hydrogen as the reducing sweep gas. The hydrogen combustion can be written as

(15) $$H_{2}+\frac{1}{2}O_{2}\to H_{2}O,$$

with the non-zero stoichiometric numbers $v_{H_{2},s}=-1,\;v_{O_{2},s}=-\frac{1}{2},\;v_{H_{2}O,s}=1$. While this process does not yield net hydrogen production, since the hydrogen produced on the feed side is consumed on the sweep side, it has been considered for previous experimental feasibility studies [5,25,26] and is used to validate the implemented water splitting mechanism in this study (c.f. Section 3.1).

Instead of hydrogen, the oxygen on the sweep side may also be consumed by partial oxidation of hydrocarbons such as methane. This allows us to couple water splitting with partial oxidation in one process to produce pure hydrogen as well as synthesis gas. The products of partial oxidation of methane are $H_{2}$ and CO in an approximate ratio of 2:1 with $H_{2}O$ and ${\mathrm{CO}}_{2}$ as by-products. The overall reactions may take several different reaction paths, and the achieved purity of the products depends on the catalyst [9,27,28]. To estimate the conversion ratios in this study, a chemical equilibrium reaction is assumed. This tends to underestimate the generation of $H_{2}O$ and ${\mathrm{CO}}_{2}$ but allows us to observe general correlations [10,29,30,31]. For this purpose, we write the partial oxidation reaction with by-products into a single reaction as

(16) $$a{\mathrm{CH}}_{4}+O_{2}\to bH_{2}+c\mathrm{CO}+dH_{2}O+e{\mathrm{CO}}_{2}$$

The non-zero coefficients $v_{i}$ required for the source terms are not fully determined by the stoichiometry and depend on the local temperature and gas composition. These are iteratively calculated for each fluid cell on the sweep side as

(17) $$v_{i}^{\left(k,l+1\right)}=v_{i}^{\left(k,l\right)}+\frac{M_{O_{2}}}{M_{i}}\left(Y_{i,eq}^{\left(k,l\right)}-Y_{i}^{\left(k,l\right)}\right),$$

where the superscript *l* denotes the current iteration for solving the fluid dynamics equations. Since based on stoichiometric considerations there are two degrees of freedom for the five coefficients $v_{i}$, Equation (17) is only used to update $v_{CO}$ and $v_{CO_{2}}$ while the other coefficients are calculated from the stoichiometric ratio at each iteration. One approach to increase the informative value of the model with regard to the production of by-products could be to assume a partial equilibrium for a single surface reaction (e.g., methane combustion) [32,33]. Further reaction steps may then be modelled via reaction rates. However, conducting a more detailed analysis of the surface reactions on the membrane would be necessary in future studies for this purpose.

During the CFD simulation, the convergence to a chemical equilibrium composition of the species on the membrane surface for the partial oxidation reaction was verified by the mean absolute error, which was calculated using *Ansys Fluent*’s internal surface integration function as follows:

(18) $$E\left(Y_{i}\right)=\frac{1}{A_{mem}}\underset{A_{mem}}{\;\int \int \;}\left|Y_{i,eq}-Y_{i}\right|dA.$$

It was found that the results of the simulation no longer changed significantly once the values for all species fell below $10^{-4}$.

### 2.2. Perfectly Mixed Reactor Model

In a previous study, we presented and validated a perfectly mixed reactor model for oxygen membrane reactors [10]. Similar to the CFD model, the driving force is calculated from the chemical equilibrium. A major difference from the CFD model is that a perfect mixture of isothermal gases in the feed and sweep gas compartments is assumed. This means that the driving force is calculated based on the outlet conditions of the reactor rather than from the actual gas compositions on the membrane surfaces. In order to show the influence of this simplified approach, the perfectly mixed reactor model is compared with the results of the CFD simulation in the present study. The complete model reads as follows:

(19) $$\begin{array}{cc} \min_{{\dot{N}}_{f/s}} & \;\;\sum_{i}{\dot{N}}_{i,f/s}\left(\frac{\Delta G_{i}^{0}\left(T\right)}{RT}+\ln\frac{P_{f/s}}{P^{0}}+\ln\frac{{\dot{N}}_{i,f/s}}{\sum_{j}{\dot{N}}_{j,f/s}}\right)\\ s.t. & \;\;\sum_{i}a_{i}\left({\dot{N}}_{i,f/s}-{\dot{N}}_{i,f/s,0}\right)\pm jA_{mem}=0\\ \; & \;\;{\dot{N}}_{f/s}\geq 0\\ \mathrm{with} & \;\;j_{k}=\left\{\begin{array}{cc} 2j_{O_{2}}, & k=O\\ 0, & k\neq O \end{array}\right.\\ \; & \;\;j_{O_{2}}=\frac{RT{\bar{\sigma }}_{amb}}{16F^{2}L}\left(\ln\frac{{\dot{N}}_{O_{2},f}}{{\dot{N}}_{O_{2},s}}+\ln\frac{\sum_{i}{\dot{N}}_{i,s}}{\sum_{i}{\dot{N}}_{i,f}}+\ln\frac{P_{f}}{P_{s}}\right) \end{array}$$

Here, ${\dot{N}}_{i,f/s}$ represents the outlet molar flow rate of species *i* on the feed/sweep side, ${\dot{N}}_{i,f/s,0}$ is the inlet molar flow rate, $\Delta G_{i}^{0}$ is its Gibbs free energy of formation at a standard state pressure, and $a_{i}$ is a vector with a length equal to the number of occurring atom types. It contains the numbers of the respective atoms of one molecule of species *i* in its row. The term $\pm jA_{mem}$, which includes the oxygen flux into the atomic balance for the chemical equilibrium calculation, is negative on the sweep side and positive on the feed side.

The required heat input for the reaction is calculated from the change in total enthalpy as

(20) $$\dot{Q}=\sum_{i}h_{i}\left({\dot{N}}_{i,f}+{\dot{N}}_{i,s}-{\dot{N}}_{i,f,0}-{\dot{N}}_{i,s,0}\right)\;,$$

where $h_{i}$ is the total enthalpy of species *i*.

The thermodynamic data for the simulations were taken from Gri-Mech 3.0 [34]. The problem is solved as a nested problem. The inner problem involves the chemical equilibrium calculations on the feed/sweep side, which are solved using Cantera 3.0 [20]. The outer problem consists of finding the oxygen flux $j_{O_{2}}$ satisfying the Wagner equation and is solved as a root-finding problem using SciPy 1.11.4 [35]. The Python script used to solve this problem is published on GitHub [36].

## 3. Results and Discussion

### 3.1. Validation

Experimental data obtained for the hydrogen production rate as well as the oxygen partial pressure at the reactor outlet from Cai et al. [5] were used to validate the water splitting mechanism implemented in the CFD simulation. In these experiments, a mixture of steam and helium was used as the feed gas, and a mixture of hydrogen and nitrogen was used as the sweep gas. The experiments involved a dual-phase membrane with an active membrane area of 0.85 cm^2^ and a thickness of 500 µm. The ambipolar conductivity required for the calculation of the oxygen flux in Equation (9) is estimated here using the specified values for the ionic conductivity $\sigma_{i}$ and the total conductivity $\sigma_{t}$ from Cai et al. [5]. Based on the definition of the ambipolar conductivity [24,37], it can be calculated as

(21) $$\sigma_{amb}=\frac{\sigma_{i}\sigma_{e}}{\sigma_{i}+\sigma_{e}}=\frac{\sigma_{i}\left(\sigma_{t}-\sigma_{i}\right)}{\sigma_{t}}\;.$$

Inserting the provided ranges for $\sigma_{i}=10–20$ S/m and $\sigma_{t}=96–944$ S/m, a lower bound for the ambipolar conductivity of 9.0 S/m and an upper bound for 19.6 S/m are obtained.

As a base case, a feed flow rate of 200 mL/min with 90% of $H_{2}O\left(g\right)$ and a sweep flow rate of 100 mL/min with $50\;\%\;H_{2}$ were used (flow rates at reference temperature 25 °C). The temperature was set to 950 °C. Since the energy source in Equation (13) cancels out when hydrogen is used as a sweep gas, the simulation remains isothermal. Starting from this base case, the parameters were varied in the simulation and compared with the experimental data.

**Figure 3:**
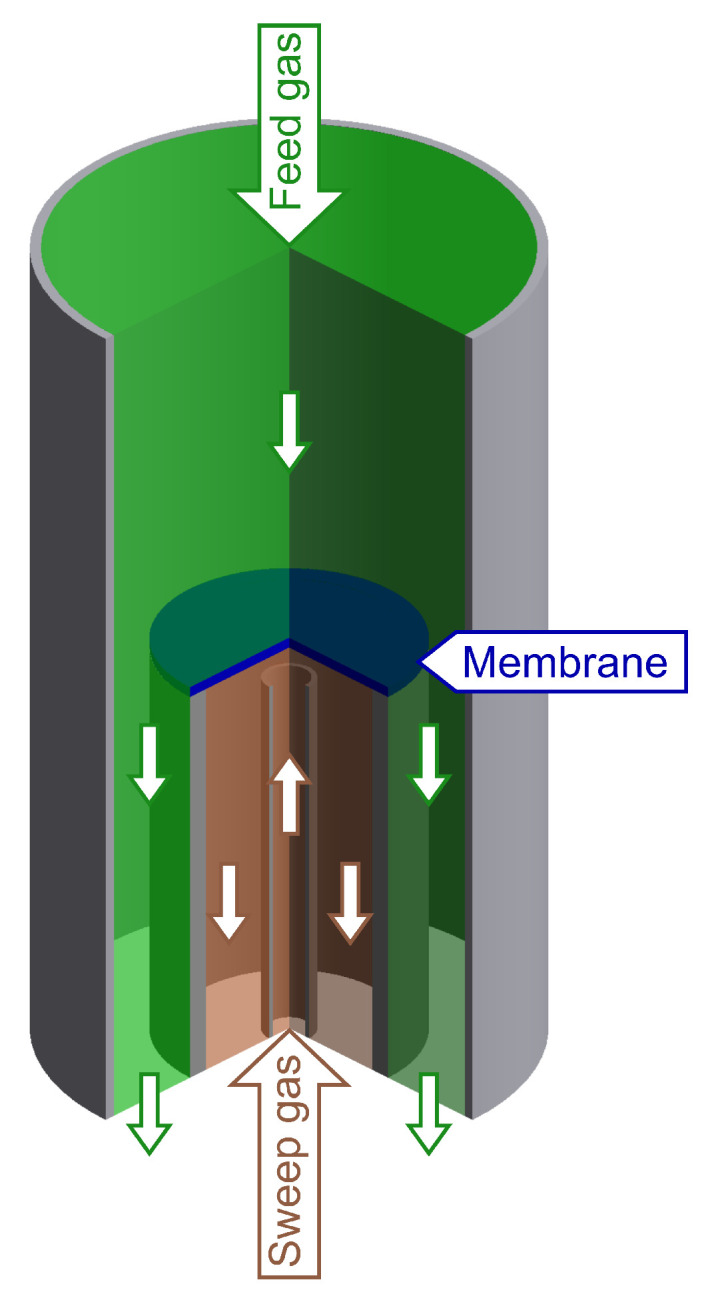
Schematic illustration of a rotational symmetric oxygen membrane reactor with a perpendicular impinged membrane.

A rotational symmetric membrane reactor with perpendicular impingement was used as schematically illustrated in Figure 3.

**Figure 4:**
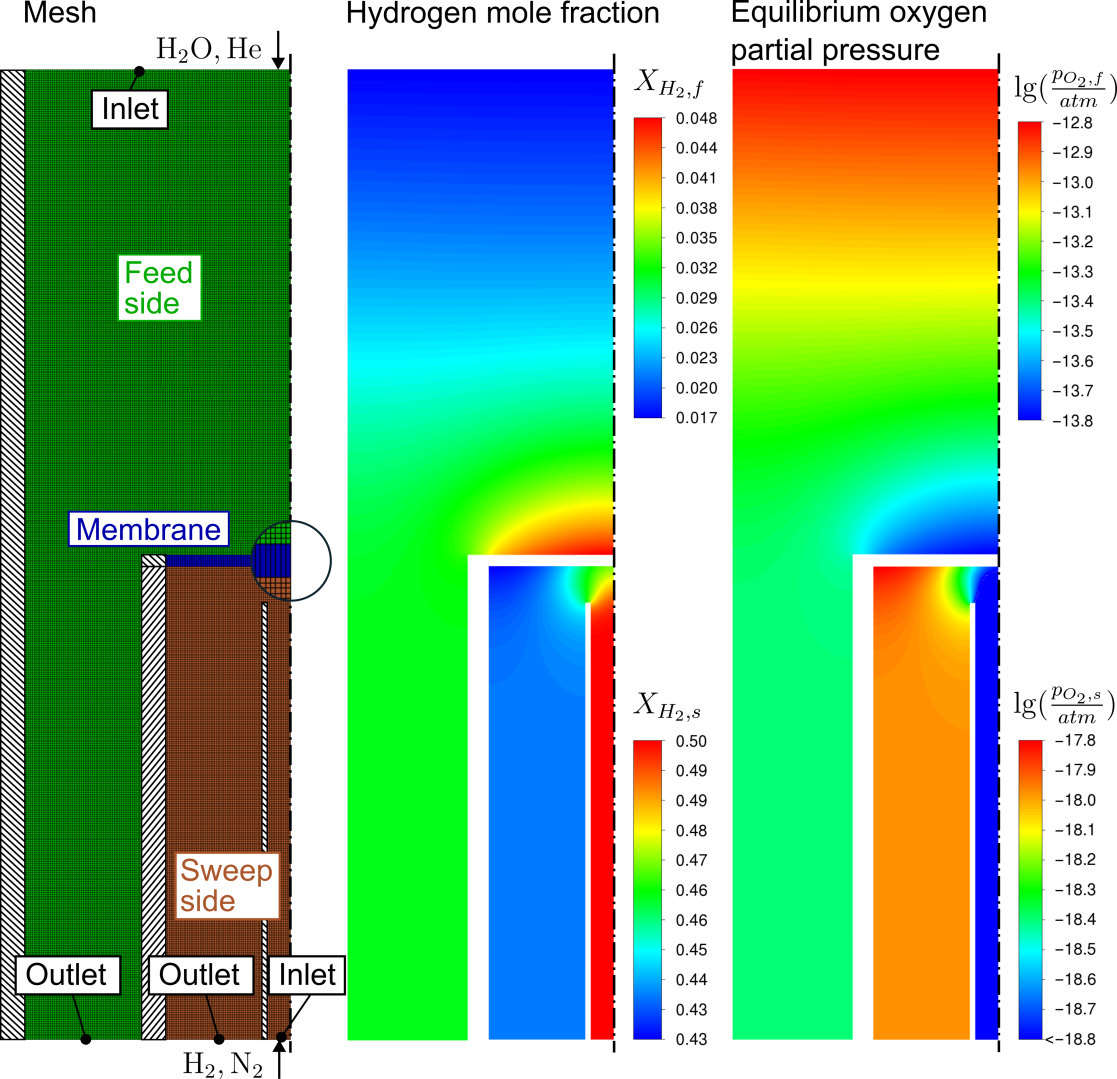
Mesh and results for the base case with $\sigma_{amb}=19.6$ S/m. A rotational symmetric mesh based on the experimental setup of Cai et al. [5] was used for the simulation.

Figure 4 shows the rotational symmetric mesh used for the simulation consisting of about 73,000 cells as well as the boundary conditions. In the simulations, the membrane was vertically impinged on the sweep side by a pipe with a diameter of 2 mm. The minimum distance of the inlets and outlets to the membrane for the simulation domain was set to 20 mm.

Using a refined mesh consisting of about 164,000 cells did not lead to any significant deviations in the evaluated results (about 0.1%). In addition to the mesh, exemplary results of the hydrogen content and the chemical equilibrium partial pressure for the base case are presented. It appears that the finite diffusion rate in the gas leads to a concentration polarization of hydrogen in the feed gas at the membrane surface. Consequently, the oxygen equilibrium partial pressure on the membrane surface is lower than at the outlet of the feed side, which can be explained by formulating the pressure equilibrium constant for the water splitting reaction as

(22) $$K_{p}=\frac{\frac{p_{H_{2}}}{P^{0}}{\left(\frac{p_{O_{2}}}{P^{0}}\right)}^{0.5}}{\frac{p_{H_{2}O}}{P^{0}}},$$

where $K_{p}$ is the pressure equilibrium constant and $P_{0}$ is the standard state pressure. By substituting the partial pressure with the mole fractions $X_{i}$ as $p_{i}=X_{i}P$, it can be shown that the chemical equilibrium oxygen partial pressure decreases with increasing $X_{H_{2}}$:

(23) $$p_{O_{2},eq}\propto {\left(\frac{X_{H_{2}O}}{X_{H_{2}}}\right)}^{2}$$

The $H_{2}$ mole fraction at the reactor outlet on the feed side is approximately 4.4% for the case shown in Figure 4. This is significantly higher than the chemical equilibrium mole fraction when no oxygen is removed, which is in the order of $10^{-3}\%$ for the given process parameters (calculated via Cantera 3.0 [20]). Since the oxygen partial pressures on the membrane surfaces differ by several orders in magnitude, the process is not limited by thermodynamics, and, thus, the $H_{2}O$ conversion could be increased by increasing the membrane area, increasing the ambipolar conductivity, or reducing the membrane thickness.

**Figure 5:**
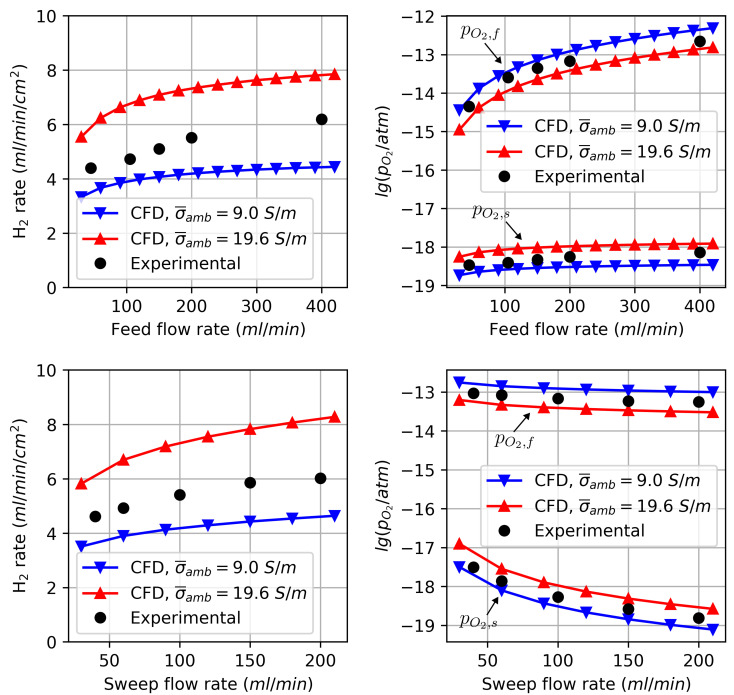

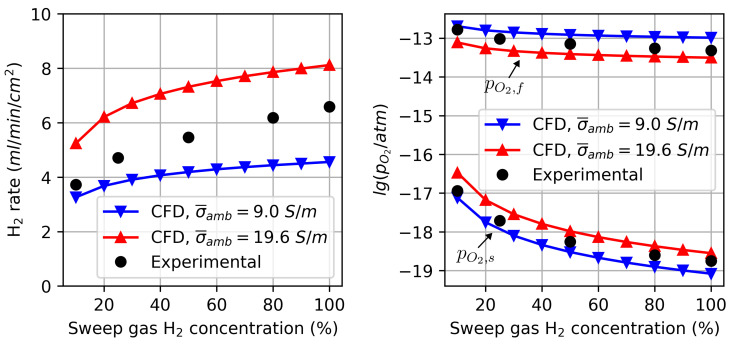
Comparison of the results obtained from the CFD model with the experimentally obtained results by Cai et al. [5]. The graphs on the left-hand side show the hydrogen production rate on the feed side. The graphs on the right-hand side show the oxygen partial pressures on the feed and sweep side, respectively. The range of the simulated data (red and blue curves) results from the uncertainty in the ambipolar conductivity.

The variation of the parameters and comparison of the hydrogen production rate as well as the oxygen partial pressure on the feed and sweep sides to experimental data are shown in Figure 5. The hydrogen production rate was determined by

(24) $$H_{2}\;\mathrm{rate}=2j_{O_{2}}$$

and converted to a volumetric flow rate using the ideal gas law at a reference temperature of 25 °C. The simulated values for the oxygen partial pressure were obtained from the outlet conditions of the reactor. Due to the uncertainty in the ambipolar conductivity ${\bar{\sigma }}_{amb}$ calculated using Equation (21), the simulation results are plotted for the upper and lower bounds, resulting in an uncertainty range. The validation demonstrates that the CFD model is in good agreement with experimental results, as they fall within this range. This also holds when varying the process parameters. When the feed flow rate is increased, the oxygen partial pressure on the feed side rises as the ratio between unconverted $H_{2}O$ to the produced $H_{2}$ increases, leading to a higher $H_{2}$ rate. Conversely, increasing the sweep flow rate or $H_{2}$ concentration reduces this ratio on the sweep side, lowering the oxygen partial pressure on the sweep side, which also increases the $H_{2}$ rate.

It should be noted that uncertainty in ambipolar conductivity is relatively high. In order to draw a more accurate comparison between experiment and simulation, it would be necessary to carry out experiments with more precisely known conductivities of the membrane material.

### 3.2. Simulation Study

#### 3.2.1. Meshes and Boundary Conditions

For the simulation study, five different CFD models were used and compared to the ideal reactor model. This was intended to analyse the impact of the geometry on the reactor performance. The CFD models are

(a) Perpendicular impinged rotational symmetric reactor with an active membrane diameter of $L_{mem}=3$ cm;
(b) A 2D reactor model in co-current flow configuration with an active membrane length of $L_{mem}=3$ cm;
(c) A 2D reactor model in a counter-current flow configuration with an active membrane length of $L_{mem}=3$ cm;
(d) A 2D reactor model in a co-current flow configuration with an active membrane length of $L_{mem}=9$ cm;
(e) A 2D reactor model in a counter-current flow configuration with an active membrane length of $L_{mem}=9$ cm.

**Figure 6:**
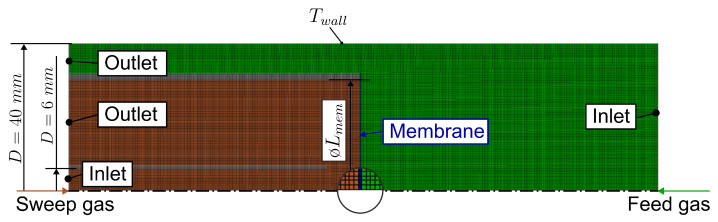
Mesh of the perpendicular impinged rotational symmetric reactor for the simulation study consisting of 71,000 cells. The geometry is schematically illustrated in Figure 3 (rotated by $90^{\circ }$).

**Figure 7:**
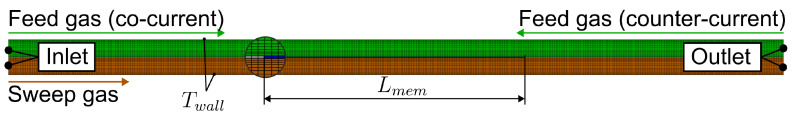
Mesh of the 2D reactor for the simulation study consisting of 18,000 cells for $L_{mem}=3$ cm and 29,000 cells for $L_{mem}=9$ cm. For the counter-current configuration, the inlet and outlet on the feed side are swapped.

The mesh and boundary conditions used for the simulation of the perpendicular impinged membrane are shown in Figure 6 and the mesh used for the 2D simulations is shown in Figure 7. Since these are relatively simple geometries with 2-dimensional meshes, no detailed mesh convergence study was conducted. However, refining the mesh did not significantly change the evaluated results (about 0.1%). In each simulation, the ratio $\frac{{\bar{\sigma }}_{amb}}{L}$ used in Equation (9) was set to $\frac{1\;S/m}{100\;\mu m}=1$ S/cm^2^, which is in the order of magnitude of ceramic oxygen transport membrane materials used for oxygen membrane reactors [5,6,38]. The values in these studies ranged from $\frac{13.5\;S/m}{1900\;\mu m}\approx 0.7$ S/cm^2^ to $\frac{19.6\;S/m}{500\;\mu m}\approx 3.9$ S/cm^2^ (estimations for ${\bar{\sigma }}_{amb}$ taken from [10]). The wall temperature was set to $T_{wall}=$ 850 °C. The thermal conductivity of the membrane was set to 3 Wm^−1^K^−1^, which is in the region of typical mixed ionic electronic conducting materials [39,40,41]. The internal emissivity of all walls was set to 0.7.

Two simulation studies were carried out with each of the models described. In the first study shown in Section 3.2.2, hydrogen was used as a sweep gas. For the second study shown in Section 3.2.3, methane was used as a sweep gas.

#### 3.2.2. Water Splitting Using Hydrogen as Sweep Gas

**Figure 8:**
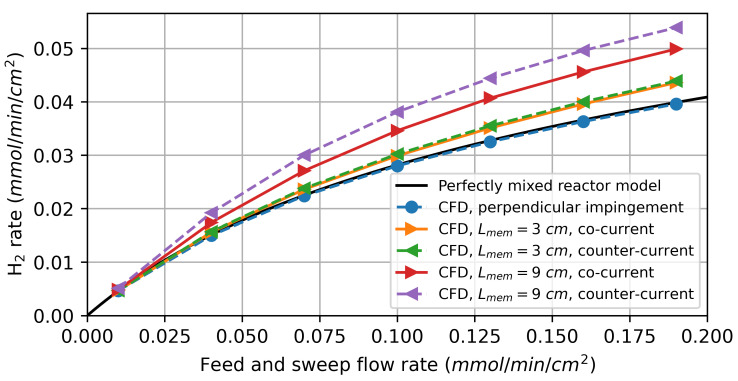
Simulation results of the $H_{2}$ production rate on the feed side for water splitting using hydrogen as a sweep gas.

In the first simulation study, pure hydrogen was used as a sweep gas. The feed and sweep flow rates were chosen to be identical and gradually increased simultaneously. Figure 8 shows the simulation results for the CFD models and the perfectly mixed reactor model. For low flow rates, the results obtained by the CFD models are nearly identical to those obtained by the perfectly mixed reactor model. This can be attributed to gas transport in the feed and sweep gas compartments being dominated by diffusion, implying that the gas composition at the membrane surface does not differ significantly from that at the reactor outlet.

**Figure 9:**
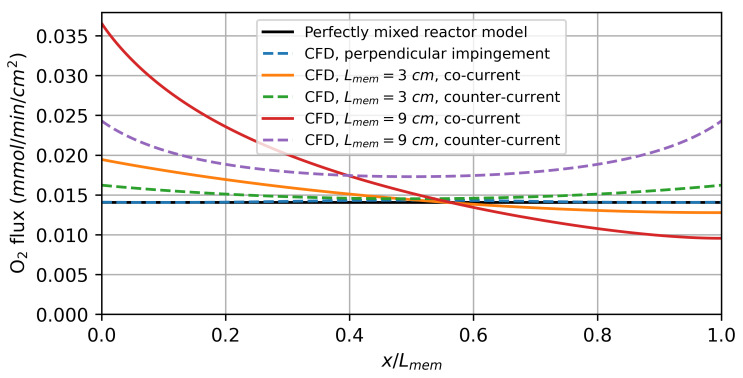
Local oxygen flux simulation results for water splitting using hydrogen as a sweep gas. The results for a feed and sweep flow rate of 0.1 mmol/min/cm^2^ are shown.

As the flow rates increase, the CFD model predicts a higher hydrogen production rate for the parallel flows along the membrane compared to the perfectly mixed reactor model. Due to the finite diffusion rate, the oxygen flux resulting from the driving force varies significantly over the reactor length, as shown in Figure 9. For the co-current flow rate, it decreases along the flow direction as the difference in the chemical potential of oxygen between the feed and sweep sides decreases. In the counter-current flow configuration, the minimum is observed at the centre of the membrane.

The varying driving force leads to a higher average oxygen flux and, thus, also a higher hydrogen production rate than predicted by the perfectly mixed reactor model, especially for the model with a 9 cm active membrane length. This can be explained by the diffusion of the reaction products against the flow direction. The diffusion of produced $H_{2}$ on the feed side against the flow direction decreases the oxygen partial pressure in the reaction chamber. On the sweep side, on the other hand, the diffusion of produced $H_{2}O$ increases the oxygen partial pressure. The net effect of this is a decrease in the average driving force. By increasing the membrane length, the ratio between the convective gas transport to the diffusive gas transport is increased, resulting in a higher overall driving force.

The counter-current flow in the reactor leads to a higher average hydrogen production rate, which is consistent with the thermodynamic analysis by Bulfin [42]. For the perpendicular impinged membrane, there is no significant deviation from the perfectly mixed reactor model, indicating that concentration polarization plays a minor role under these conditions.

#### 3.2.3. Water Splitting Using Methane as Sweep Gas

The estimation of a reasonable order of magnitude for feed and sweep gas flow rates for the base case considered in the simulations was conducted using the perfectly mixed reactor model. This was performed under the condition that a ${\mathrm{CH}}_{4}$ conversion of 95% and a $H_{2}O$ conversion of 25% should be achieved. These were evaluated as

(25) $${\mathrm{CH}}_{4}\;\mathrm{conversion}=\frac{{\dot{N}}_{CH_{4},s,0}-{\dot{N}}_{CH_{4},s}}{{\dot{N}}_{CH_{4},s,0}}$$

and

(26) $$H_{2}O\;\mathrm{conversion}=\frac{{\dot{N}}_{H_{2}O,f,0}-{\dot{N}}_{H_{2}O,f}}{{\dot{N}}_{H_{2}O,f,0}}$$

**Figure 10:**
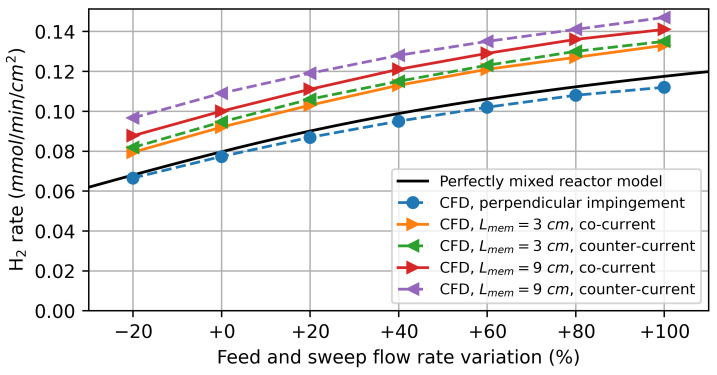
Simulation results of the $H_{2}$ production rate on the feed side for water splitting using methane as a sweep gas. The flow rate variation refers to the percentage variation from the base flow rates (base feed flow rate: 0.319 mmol/min/cm^2^ and base sweep flow rate: 0.080 mmol/min/cm^2^).

**Figure 11:**
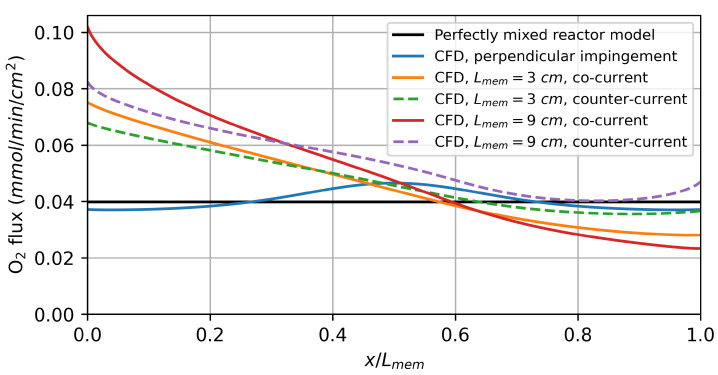
Local oxygen flux simulation results for water splitting using methane as a sweep gas. The simulation results for the base flow rates are shown (base feed flow rate: 0.319 mmol/min/cm^2^ and base sweep flow rate: 0.080 mmol/min/cm^2^).

A feed flow rate of 0.319 mmol/min/cm^2^ and a sweep flow rate of 0.080 mmol/min/cm^2^ were obtained. These flow rates were then simultaneously increased in the simulations. Results for the mean hydrogen production rate and the local oxygen flux are shown in Figure 10 and Figure 11, respectively. Here, hydrogen production again refers solely to the hydrogen produced by water splitting on the feed side. Similar to the previous simulations with hydrogen as a sweep gas, the highest hydrogen production is achieved by the model with the 9 cm long membrane in the counter-current flow configuration. In the case of perpendicular impinged flow, the highest $O_{2}$ flux is observed in the centre of the membrane, where it is impinged by the sweep gas. Its average hydrogen production is slightly lower at higher flow rates than in the perfectly mixed reactor. This indicates that the concentration polarization at the membrane surface reduces the hydrogen production for these flow conditions.

Table 1 shows the relative change in hydrogen production rate simulated by the CFD model compared to the perfectly mixed reactor model, demonstrating the reactor geometries’ significant impact on the hydrogen production rate.

**Figure 12:**
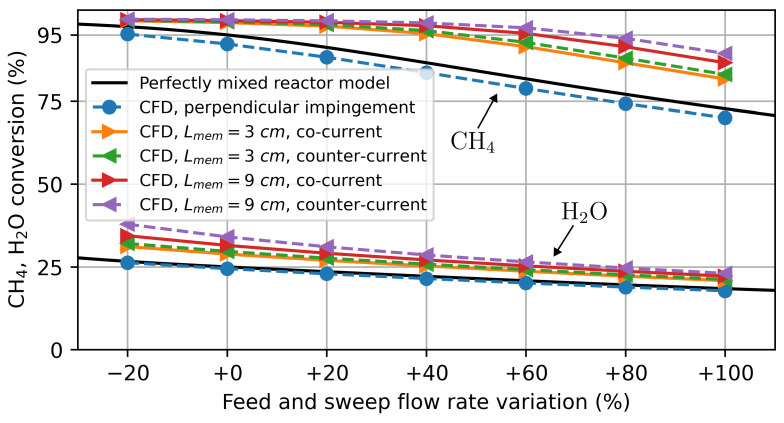
${\mathrm{CH}}_{4}$ and $H_{2}O$ conversion simulation results for water splitting using methane as a sweep gas. The flow rate variation refers to the percentage variation from the base flow rates (base feed flow rate: 0.319 mmol/min/cm^2^ and base sweep flow rate: 0.080 mmol/min/cm^2^).

The achieved ${\mathrm{CH}}_{4}$ conversion (Equation (25)) on the sweep side and $H_{2}O$ conversion (Equation (26)) on the feed side are shown in Figure 12. According to the CFD model, the reactors with parallel flows along the membrane could be operated with significantly higher sweep and feed gas flows than estimated by the ideal reactor model while still achieving the targeted conversion rates of 25% for $H_{2}O$ and 95% for ${\mathrm{CH}}_{4}$. The flow rates for the 9 cm long reactor may, thus, be increased by around 70% in the counter-current flow configuration.

**Figure 13:**
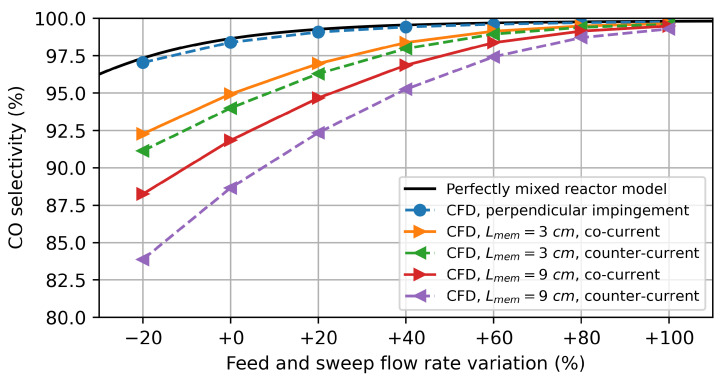
CO selectivity simulation results for water splitting using methane as a sweep gas. The flow rate variation refers to the percentage variation from the base flow rates (base feed flow rate: 0.319 mmol/min/cm^2^ and base sweep flow rate: 0.080 mmol/min/cm^2^).

In Figure 13, the CO selectivity defined as

(27) $$\mathrm{CO}\;\mathrm{selectivity}=\frac{{\dot{N}}_{CO}}{{\dot{N}}_{CH_{4},0}-{\dot{N}}_{CH_{4}}}$$

is depicted. It should be noted once again that the CO selectivity is typically overestimated within the chemical equilibrium calculation. Nevertheless, according to chemical equilibrium, a CO selectivity close to 100% is achievable if a 95% methane conversion is aimed for. At lower flow rates, the CO selectivity decreases, as larger quantities of ${\mathrm{CO}}_{2}$ are produced when the methane conversion is close to 100%.

**Figure 14:**
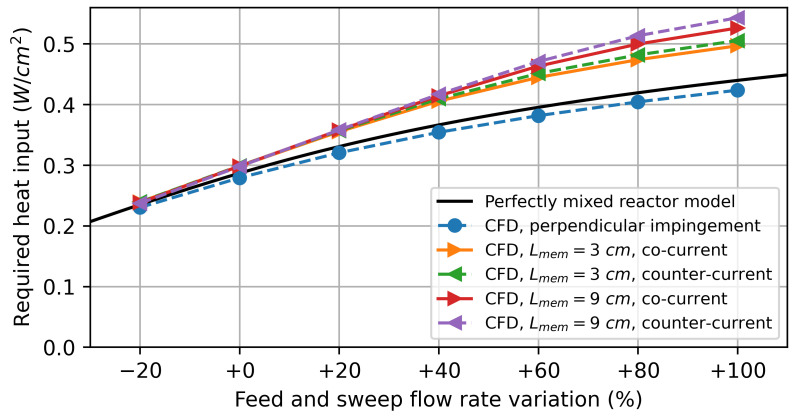
Required heat input for water splitting using methane as a sweep gas. The flow rate variation refers to the percentage variation from the base flow rates (base feed flow rate: 0.319 mmol/min/cm^2^ and base sweep flow rate: 0.080 mmol/min/cm^2^).

**Figure 15:**
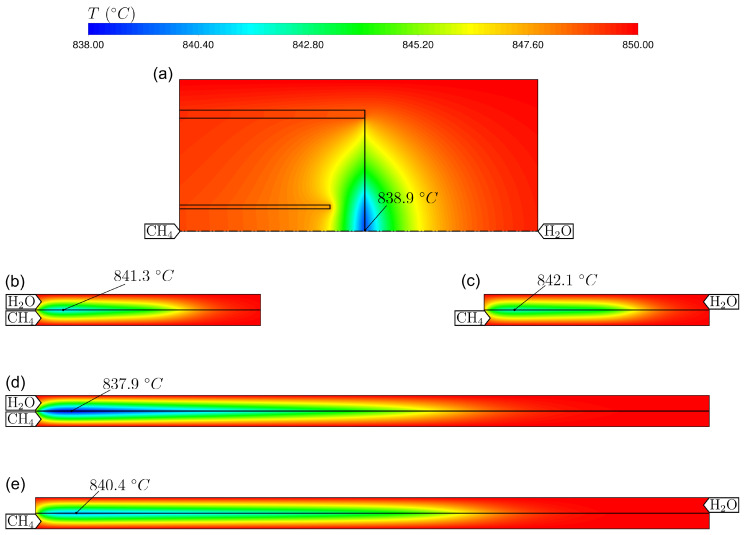
Simulated temperature distribution for water splitting using methane as a sweep gas. The results for the base flow rates are shown. For the simulations with parallel flows along the membranes, only the active membrane length regions are shown. The cases (**a**–**e**) refer to the CFD models described in Section 3.2.1.

Finally, the temperature distribution inside the reactor can also be evaluated using the CFD model. Figure 14 shows the required heat input for the reactions, which are net endothermic. For the perfectly mixed reactor model, this was calculated using Equation (20), while for the CFD models, the net heat flux through the boundaries was evaluated. Due to the local energy source term implemented in the CFD simulation, a cooling of the membrane can be observed, as shown in Figure 15. The maximum temperature drop in the counter-current flow operation is lower than in the co-current flow operation. This can be explained by the fact that in counter-current flow, the oxygen transport is more evenly distributed over the length of the reactor as demonstrated in Figure 11. It should be noted here that the effect of the temperature drop in the reactor on the ambipolar conductivity was not considered in this study. For the design of larger reactors in which significant temperature differences may occur, this can be taken into account by the temperature-dependent modelling of the ${\bar{\sigma }}_{amb}$ in Equation (9).

## 4. Conclusions

The proposed CFD modelling approach to water splitting inside oxygen membrane reactors agreed well with the experimental results, in which hydrogen was used as a reducing gas on the sweep side to establish the gradient in chemical potential, as shown in the validation study. The approach allows for the hydrogen production rate to be determined from the membrane and process parameters by implementing the Wagner equation in a CFD simulation. In addition to the considered case using hydrogen as a sweep gas, the water splitting model was coupled with a chemical equilibrium calculation for partial oxidation to estimate the performance of water splitting when methane is used as a sweep gas. By considering the reaction enthalpy in this process, the temperature distribution in the reactor can also be investigated using the CFD model.

The simulation study indicates that the performance of oxygen membrane reactors is heavily influenced by the geometrical design. For reactors with parallel flows along membranes, a higher oxygen flux was observed in the CFD simulations than the predicted values by the perfectly mixed reactor model, as a higher average driving force can be achieved due to the concentration profile along the membrane surfaces. This effect can be increased by increasing the reactor’s length. In this study, the counter-current flow configuration resulted in an increased hydrogen production rate compared to the co-current flow configuration, especially when the reactor length was increased. Another advantage of the counter-current flow configuration was that the oxygen flow was distributed more evenly over the length of the membrane, resulting in a lower maximum temperature drop.

By extending the model, thermal stresses in the membrane and the effects of temperature-dependent membrane properties on performance could be investigated in a reactor design. Furthermore, in future studies, the limitations of the presented model should be investigated. Especially for membranes with high oxygen permeability, the rates of the surface reactions, including all steps from $H_{2}O$ to the absorbed oxygen ions, may limit the oxygen transport. Since the model was validated for a comparatively small membrane surface of 0.85 cm^2^, the applicability to larger membrane surfaces should be further investigated experimentally. Experiments in which the ambipolar conductivity is known precisely are required to quantify the error of the model. In addition, for a more accurate modelling of the partial oxidation of methane, the surface reactions of this process should be investigated. Based on this, a multi-step reaction mechanism could be implemented to accurately predict the species components in the sweep gas.

## Figures and Tables

**Table 1:** Relative change in hydrogen production rate simulated by the CFD model compared to the perfectly mixed reactor model (c.f. Figure 10) when using methane as the reducing gas. The results for the models (a)–(e) described in Section 3.2.1 for the base flow rates are shown.

	Perpendicular Impinged Membrane (a)	Parallel Flow, *L_mem_* = 3 cm (b,c)	Parallel Flow, *L_mem_* = 9 cm (d,e)
Co-current	−3%	+15%	+25%
Counter-current		+18%	+36%

## Data Availability

The code used for the perfectly mixed reactor model simulations in the study is openly available at GitHub at https://github.com/KabitGit/Ideal-Equilibrium-Oxygen-Membrane-Reactor). Simulated raw data will be made available by the authors on request.

## Abbreviations

The following abbreviations are used in this manuscript:

CFD	Computational fluid dynamics
UDF	Userdefined function
